# Cutaneous Manifestation of a Rare Haematological Malignancy: A Case Report of the Presentation, Diagnosis, and Management of Blastic Plasmacytoid Dendritic Cell Neoplasm

**DOI:** 10.7759/cureus.87604

**Published:** 2025-07-09

**Authors:** Syeda Liaba Hassan, Danyal Elahi Chatha

**Affiliations:** 1 Medicine, Russells Hall Hospital, Dudley, GBR; 2 Haematology, Russells Hall Hospital, Dudley, GBR

**Keywords:** allogenic bone marrow transplant, blastic plasmacytoid dendritic cell neoplasm (bpdcn), cd123, cd4+cd56+, dermpath, skin manifestations, superficial skin lesions

## Abstract

Blastic plasmacytoid dendritic cell neoplasm (BPDCN) is a rare and aggressive hematological malignancy characterized by the proliferation of abnormal plasmacytoid dendritic cells. These cells infiltrate the skin and other organs during malignancy, leading to the development of violaceous skin nodules. We report a case of a middle-aged patient who presented to a secondary hospital with persistent skin lesions unresolved by primary care management and corticosteroids. A surgical excision and biopsy in secondary care revealed malignant plasmacytoid activity diffusely positive for Cluster of Differentiation (CD)4, CD123 and CD56 alongside a high Ki-67 proliferation index.

A hyperfractionated cyclophosphamide, vincristine sulfate, doxorubicin hydrochloride (Adriamycin), and dexamethasone (Hyper-CVAD) chemotherapy regimen was initiated for this patient, which resulted in a significant reduction in lesion size and an initial remission. Central nervous system (CNS) prophylaxis with intrathecal methotrexate was provided following chemotherapy to prevent CNS relapse while awaiting allogenic bone marrow transplantation.

This case highlights the importance of considering BPDCN in patients with persistent or unusual skin lesions and highlights the critical role of early biopsy and immunohistochemistry in achieving an accurate diagnosis. Moreover, it emphasizes the need for a multidisciplinary team (MDT) approach to ensure optimal management and improve prognosis and patient care with this malignancy.

## Introduction

Blastic plasmacytoid dendritic cell neoplasm (BPDCN) is a rare and often aggressive hematological malignancy, characterized by an abnormal proliferation of precursor plasmacytoid dendritic cells [1]. These plasmacytoid cells physiologically produce type 1 interferons in the body to maintain immunity, especially against viral antigens [2].  However, in malignant states, these plasmacytoid dendritic cells (pDCs) acquire mutations that enable them to infiltrate the skin, lymph nodes, and other organs [3]. This skin infiltration leads to the development of violaceous skin nodules, which are often the first sign of malignancy in many patients [4].    

The primary aim of this case report is to enable healthcare professionals to identify the cutaneous manifestations of BPDCN, thereby facilitating faster recognition, evaluation, and management of the malignancy. This case report follows a 64-year-old male patient from the West Midlands who was referred to a district general hospital with widespread, discrete nodular skin lesions spanning his body. His initial cutaneous presentation with BPDCN and absence of constitutional features of malignancy, coupled with the disease’s quick progression and poor prognosis highlights the importance of considering BPCDN in patients with unusual or persistent skin lesions that remain unresponsive to treatment. 

## Case presentation

This case report follows a middle-aged male patient who first presented to primary care with a growing lesion on his anterior chest wall, which was persistent without significant pain. It was a raised and nodular 3 cm X 2 cm lesion, with underlying erythema. No history of fever, night sweats, weight loss, recurrent infections, anemia, abnormal bleeding, mouth ulcers, joint pain or swelling, and respiratory or gastrointestinal compromise was reported. Initial blood investigations, including a complete blood count, were within normal limits with no indication of pancytopenia, neutropenia or other abnormalities (Table 1).

**Table 1:** Blood test results from the patient's first presentation to secondary care; the only abnormality of note was a slightly raised CRP level

Tests	Reference ranges	Blood levels at initial presentation
CRP (mg/L)	0.5-5.0	10
Hemoglobin (g/L)	130-180	143
White blood cell (X 10^9^/L)	4.0-11.0	4.4
Platelet count (X 10^9^/L)	150-450 10	172

The patient had a past medical history of type 2 diabetes mellitus, hypertension and chronic kidney disease, for which he was on regular medications provided by his general practitioner. He also had a myocardial infarction in his 30s, from which he had fully recovered with no long-lasting complications at presentation. 

Based on this history and the isolated lesion, initial treatment with topical steroids for a presumed benign swelling was provided for two weeks, which was ineffective. Since the lesion persisted despite steroid treatment, the patient presented to secondary care where the lesion was suspected to be a simple abscess and an incision and drainage (I&D) under general anesthesia was arranged. The excised lesion was sent for a biopsy and histopathological analysis. The patient was sent home following the uncomplicated I&D with a seven-day course of oral flucloxacillin. 

**Figure 1:**
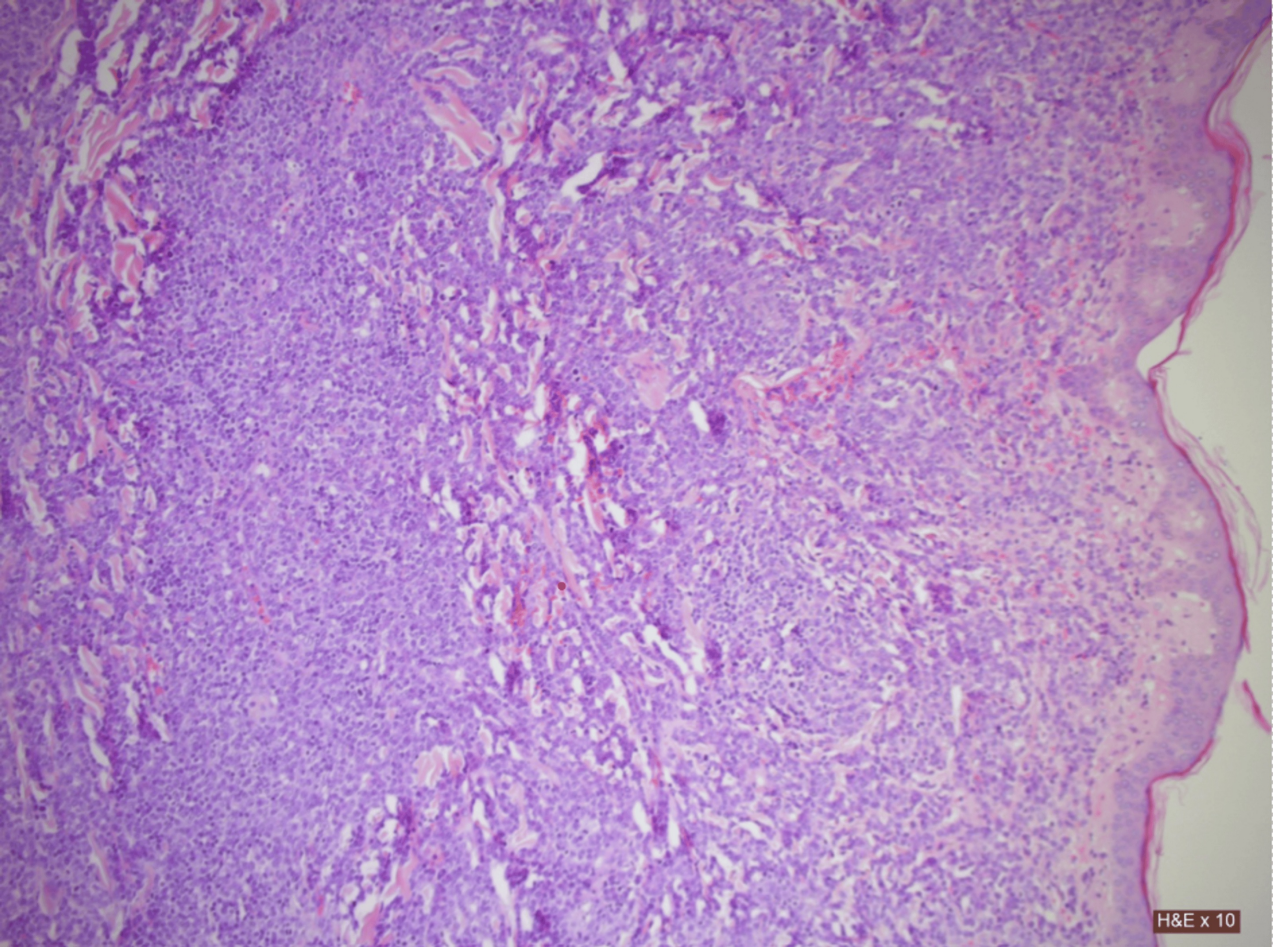
Low-power magnification of a hematoxylin and eosin (H&E) stained tissue sample showing dermal involvement with dense cellular infiltrate

The skin biopsy results yielded multiple abnormalities consistent with malignant activity (Figure 1).

The dermal infiltrate was dense and consisted of atypical monomorphic cells with vesicular, irregular nuclei extending to the subcutaneous fat and adrenexal tissue. The infiltrate exhibited a sheet-like growth pattern, with cells exhibiting scant cytoplasm. A varied chromatin structure with frequent plasmacytoid activity showing both fine and coarse chromatin was also observed. 

**Figure 2:**
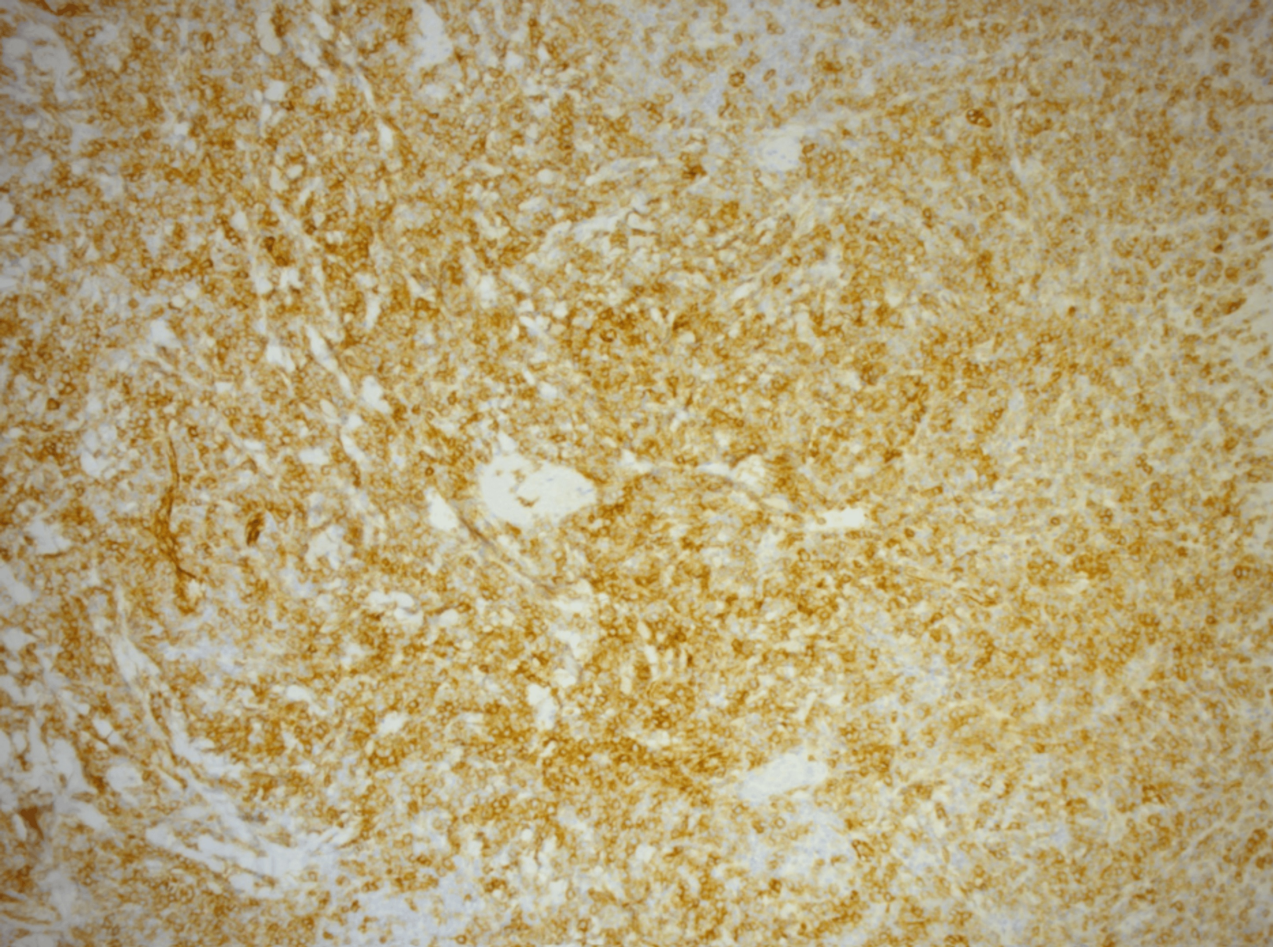
Immunohistochemical (IHC) staining of the skin tissue The brown coloration with diaminobenzidine (DAB) stain represents strong CD123 expression, with diffuse staining involving a large portion of the sample

An immunohistochemical analysis yielded diffusely positive Cluster of Differentiation (CD)4, CD123, (Figure 2) and CD56, highly suggestive of a malignancy from a plasmacytoid dendritic cell line.

A Ki-67 proliferation index of 70% was noted, indicating high mitotic activity, consistent with the aggressive disease profile. The panel included markers such as CD10, CD21, CD23, and AE1/AE3, which is an anti-epithelial cytokeratin antibody cocktail. Also included were Melanoma Antigen Recognized by T-cells 1 (Melan-A), CD34, CD117, CD138, and CD38. Additional markers were Cyclin D1, a cell cycle regulator protein; Multiple Myeloma Oncogene 1 (MUM1 or IRF4); Anaplastic Lymphoma Kinase 1 (ALK); Terminal deoxynucleotidyl transferase (TdT); B-cell Lymphoma 6 protein (BCL6); and IgM. All of these were negative, ruling out other potential hematological malignancies. This dermatopathological analysis strongly suggested a blastic plasmacytoid malignancy and warranted an urgent hematology referral, following which the patient was called into the rapid access clinic at our hospital. The diagnosis, further management, and disease prognosis were explained to him. 

**Figure 3:**
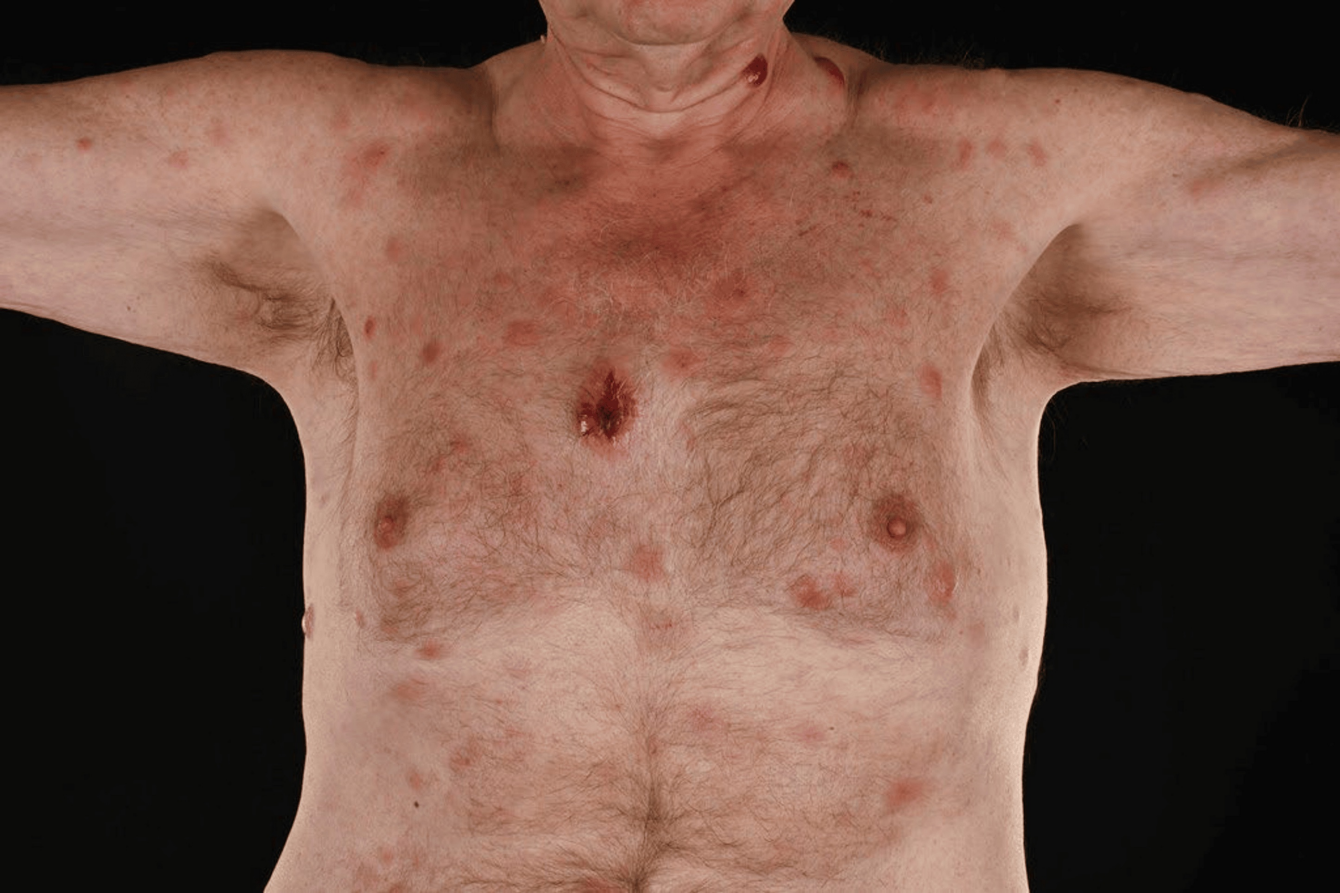
Multiple cutaneous lesions on the anterior chest wall, with the initial large central lesion The large central lesion had a crater, secondary to the incision and drainage. The lesions can also be seen extending into the arms bilaterally.

In this appointment, it was also evident that the patient's clinical picture had progressed rapidly, with multiple lesions spanning his anterior and posterior trunk, and arms and legs bilaterally (Figure 3).

**Figure 4:**
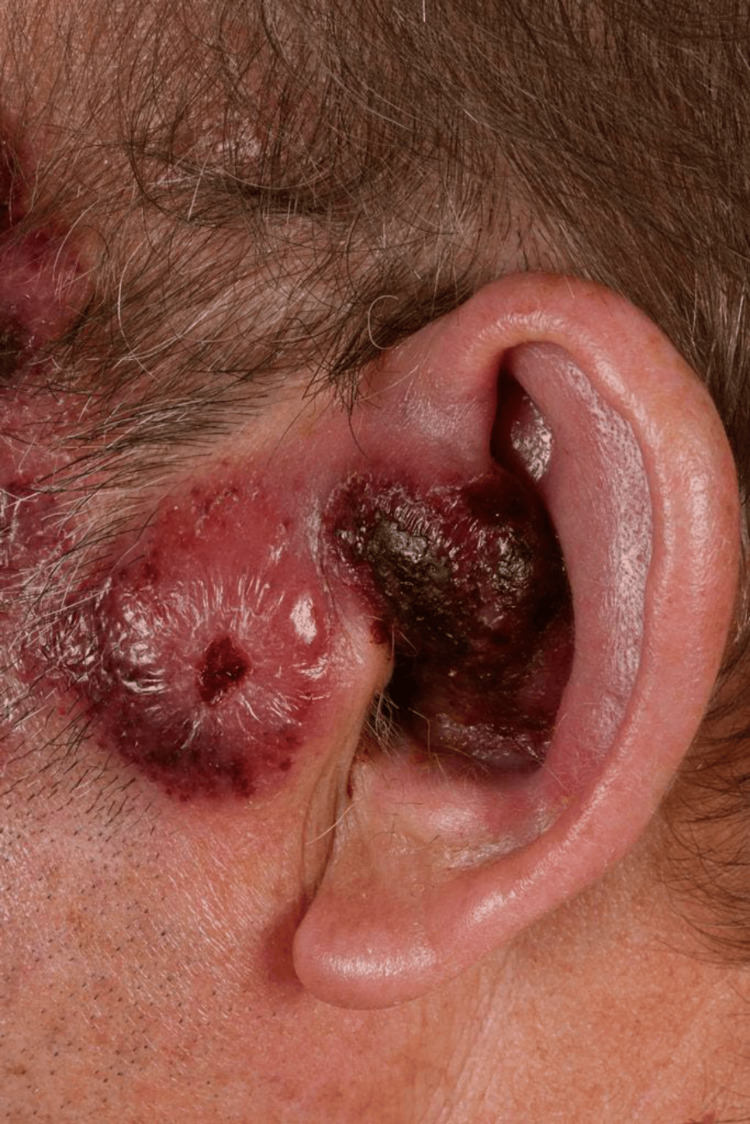
Left auricular lesion which extended into the ear canal and progressed to occlude it, as shown here

Most evident was a lesion on the left side of his face which had grown and advanced to occlude his left external auditory canal (Figure 4).

No palpable peripheral lymphadenopathy or hepatosplenomegaly was identified. 

A positron emission tomography (PET) scan revealed extensive fluorodeoxyglucose (FDG) uptake in multiple soft tissue cutaneous lesions, reflecting high metabolic activity and widespread disease burden. Bone marrow aspirate and trephine (BMAT) showed mild dysplastic changes in erythroid and myeloid lineages with slight left shift but no excess CD123-positive cells. Two percent blasts were noted in trephine, ensuring no bone marrow involvement. 

Treatment

BPDCN proliferates rapidly, which warrants prompt recognition of the malignancy and treatment initiation. Even within hematology, this cancer is exceedingly rare and makes up only 0.5% of all hematological cancers [3]. As a result, little standardized treatment exists, with most initial chemotherapy based on institutional preference [3,4].

For this patient, advice was sought from a tertiary center in the region and initial treatment with hyper-CVAD was initiated. The treatment combines cyclophosphamide, vincristine, doxorubicin hydrochloride and dexamethasone into one fractionated, chemotherapy regime. While primarily used for acute lymphoblastic leukemia, good disease response and remission has been seen in BPDCN. It is primarily used to bridge the treatment gap while a donor for a curative allogenic transplant can be found [3]. 

Hyper-CVAD is a 14-day intensive course which depletes cell counts, typical of chemotherapy. The patient was started on prophylactic ciprofloxacin while in our isolation unit, as we anticipated a drop in white cell and neutrophil counts during the chemotherapy regime. We maintained a low threshold for initiating IV antibiotics for neutropenic sepsis in the event of any new rise in CRP levels or temperature spikes as an inpatient. We also started him on acyclovir and co-trimoxazole for viral and pneumocystis jirovecii pneumonia (PJP) prophylaxis, respectively. Allopurinol was initiated first for tumor lysis prophylaxis, and then switched to rasburicase as the lesions rapidly responded to the treatment. Chlorohexidine mouthwash and benzylamine oral spray were also prescribed to help prevent mucositis commonly encountered by patients undergoing this intensive regime.

For this patient, two cycles of hyper-CVAD were initiated on separate occasions with an excellent overall response. While hyper-CVAD showed a promising initial response, it is not curative, and a high relapse rate has been observed previously where patients presented similarly [4]. Hyper-CVAD followed by allogenic transplantation remains to be the best therapeutic approach currently [4,5].  

An allogenic bone transplant (allo-BMT) uses a healthy donor's bone cells to induce proliferation of the host's viable bone marrow in a process called engraftment [6] and is used mainly after disease remission in the context of hematological cancers. This process can be physically taxing on the recipient's body and extensive organ function analysis needs to be done before initiating it. Our initial workup qualified our patient for the allogenic transplant, and he was listed and screened for it at a tertiary center in the region.  

Another characteristic of this malignancy is the high rate of relapse in the central nervous system (CNS) and CNS prophylaxis is crucial for patients undergoing treatment for this malignancy [7]. While awaiting transplantation, our patient was given intrathecal methotrexate in between hyper-CVAD cycles.

Outcome 

Our patient showed a drastic and visible response to the first cycle of chemotherapy, with the most notable lesion occluding the left auditory canal falling off spontaneously on day two of chemotherapy. Other lesions showed a marked improvement as well, with most lesions on his arms and chest reducing in size over the next few days. 

Our patient had some minor medical issues during this admission, particularly frequent episodes of hyperglycemia post steroid therapy, which we managed with as-needed fast-acting subcutaneous insulin injections. Our patient never had a spike in temperature during this admission. However, he had an episode of rigors with a spike in inflammatory markers on day nine of the cycle (Table 2), for which he was treated promptly with broad-spectrum IV antibiotics to help manage any potential infection.

**Table 2:** Blood results from our patient’s first inpatient stay in hospital, for the first 14-day cycle of hyper-CVAD Day+1: day one after chemotherapy cessation; Day+3: day three after chemotherapy cessation; Hyper-CVAD: hyperfractionated cyclophosphamide, vincristine sulfate, doxorubicin hydrochloride (Adriamycin), and dexamethasone The patient’s blood results were closely monitored for tumor lysis syndrome for the first few days of chemotherapy, as the lesions continued to shrink rapidly. As the course of the treatment progressed, our patient became deeply neutropenic. However, he recovered these counts swiftly and was discharged home after his neutrophil counts recovered.

Tests	Reference range	Day 2	Day 3	Day 4	Day 5	Day 7	Day 9	Day 11	Day 13	Day +1	Day +3
												Urea (mmol/L)	2.5-7.8	6.8	10.9	13.3	11.4	12.2	8.1	8.8	7.8	10.60	9.5
Creatinine (mmol/L)	64-111	129	113	109	92	84	109	100	92	91	84
Adjusted Calcium (mmol/L)	2.20-2.60	2.41	2.27	2.18	2.19	2.08	2.11	2.15	2.12	2.13	NA
Phosphate (mmol/L)	0.80-1.50	1.37	1.24	1.04	0.98	0.96	0.73	0.76	NA	NA	NA
CRP (mg/L)	0.5-5.0	17	18	10	7	2	206	188	87	27	14
Uric acid (mmol/L)	200-430	NA	347	<18	<18	<18	66	159	NA	NA	NA
Haemoglobin (g/L)	130-180	133	124	128	115	120	116	114	87	96	91
White blood cell count (X 10^9^/L)	4.0-11.0	1.8	2.00	4.20	8.80	6.60	1.40	0.10	0.10	0.50	3.50
Platelet count (X 10^9^/L)	150-450	188	112	134	126	114	43	23	15	31	83
Neutrophil count (X 10^9^​​​​​​​/L)	2-7.5	1.12	1.12	3.23	7.25	6.14	1.31	0.03	0.03	0.33	1.41

No new lesions were observed during the patient's stay in the hospital, and he was successfully discharged home following count recovery.  

During our patient's second admission for hyper-CVAD, he tolerated chemotherapy well, but developed marked weight gain and dyspnea mid-cycle. Doxorubicin, known to be a cardio-toxic component of hyper-CVAD, especially in cumulative doses, was suspected as the cause [8]. An echocardiogram revealed severely impaired left ventricular systolic dysfunction and a left ventricular ejection fraction (EF) of 25-30%, a significant reduction from a baseline of about 60%. This new acute heart failure could have been a possible deterrent to allo-BMT; however, after several weeks of heart failure management as per the cardiology team in the hospital, his EF improved to 55%, close to baseline. As an inpatient, he had a further episode of rigors and pyrexia on day 13 of treatment (Table 3), with a sharp CRP rise while neutropenic, for which he was treated with IV tazocin and IV gentamicin as per the neutropenic sepsis guidelines.

**Table 3:** Details of blood results from our patient’s second admission where day one signifies the first day of the hyper-CVAD chemotherapy regime Day+1: day one after chemotherapy cessation; Day+3: day three after chemotherapy cessation; Day+5: day five after chemotherapy cessation; Hyper-CVAD: hyperfractionated cyclophosphamide, vincristine sulfate, doxorubicin hydrochloride (Adriamycin), and dexamethasone Tumor lysis blood cell levels were not actively monitored for this admission, due to resolution of all previous cutaneous lesions with no disease recurrence. The patient remained deeply neutropenic throughout most of the treatment and was given filgrastim injections to stimulate rapid count recovery, following which he was safely discharged home.

Tests	Reference ranges	Day 1	Day 3	Day 5	Day 7	Day 9	Day 11	Day 13	Day +1	Day +3	Day +5
CRP (mg/L)	0.5-5.0	24	7	118	113	137	120	153	59	38	31
Hemoglobin (g/L)	130-180	91	73	104	74	77	79	92	84	88	87
												White blood cell counts (X10^9^/L)	4.0-11.0	5.90	8.80	10.20	2.30	0.10	0.00	0.30	6.40	24.70	22.90
Platelet counts (X10^9^/L)	150-450	126	97	52	12	10	17	32	72	111	155
Neutrophil counts (X10^9^​​​​​​​/L)	2-7.5	5.47	8.64	10.13	3.62	0.06	0.00	0.18	5.47	21.71	20.36

The rest of his stay was uneventful until count recovery. 

**Figure 5:**
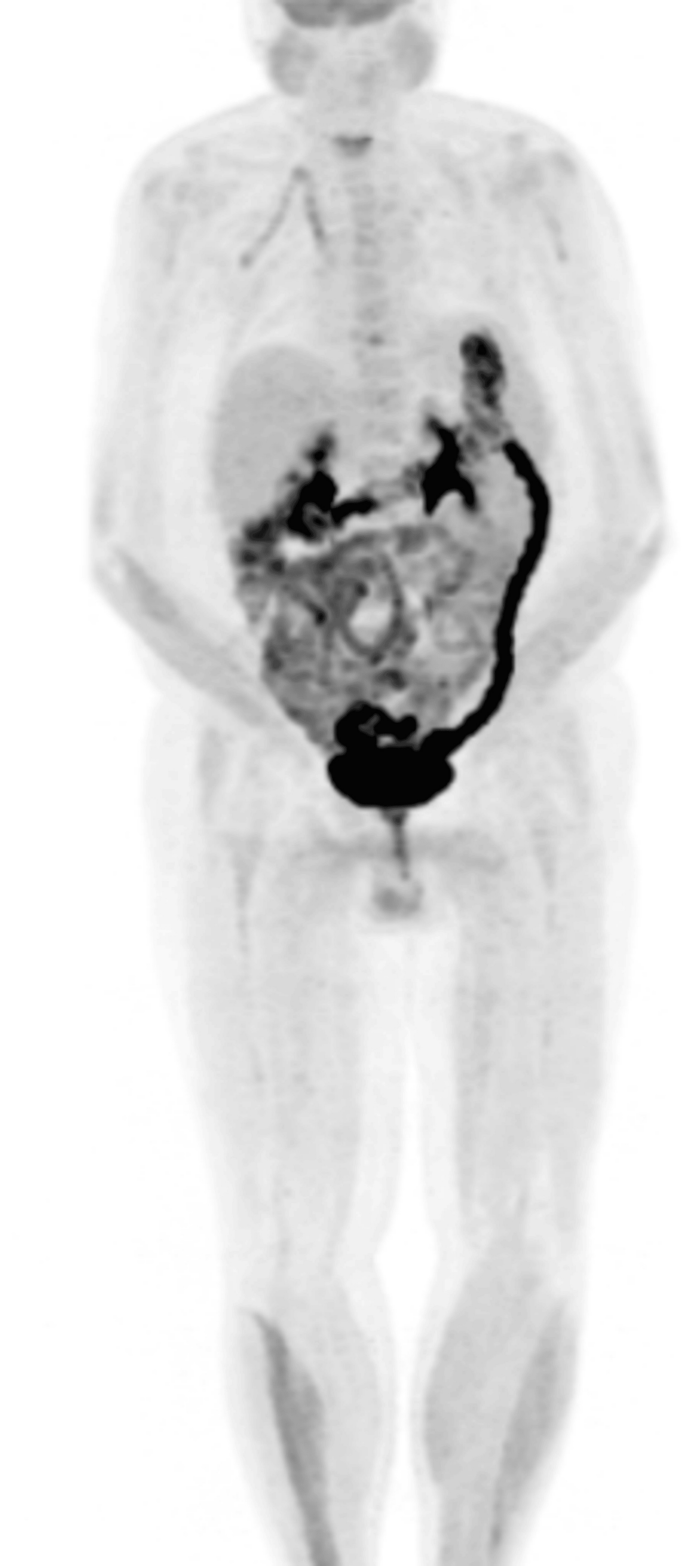
Repeat PET scan after the completion of the two hyper-CVAD chemotherapy cycles FDG: fluorodeoxyglucose; Hyper-CVAD: hyperfractionated cyclophosphamide, vincristine sulfate, doxorubicin hydrochloride (Adriamycin), and dexamethasone There was a marked difference from the previous PET scan with no avid FGD uptake, indicating good disease response to chemotherapy

**Figure 6:**
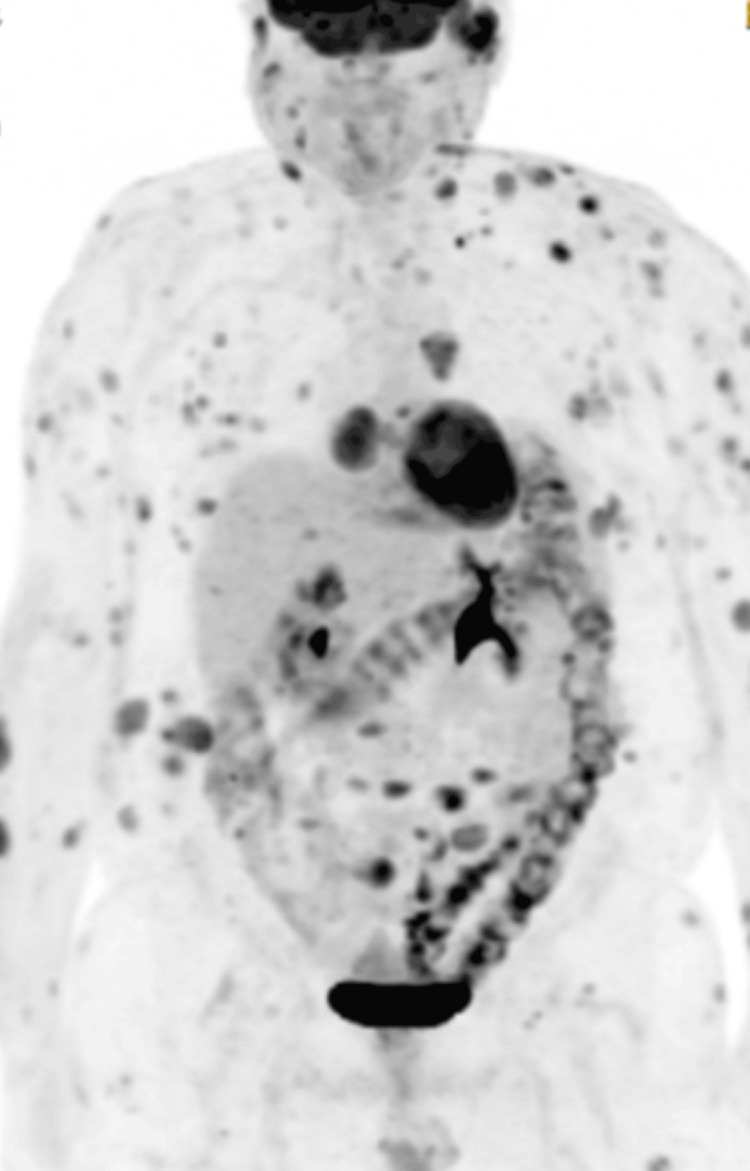
Initial PET scan showing widespread FGD uptake, concentrated primarily around the trunk and upper extremities FGD: fluorodeoxyglucose

Following discharge, and after completing second cycle of hyper-CVAD, we followed him up fortnightly with repeat BMATs which continued to show disease remission with no infiltration to bone marrow. A repeat PET scan after chemotherapy showed marked resolution of the cutaneous lesions and minimal fluorodeoxyglucose (FGD) uptake (Figure 5), a significant improvement from the initial PET scan before treatment (Figure 6).

This remained the case for all further PET scans as well. 

Our patient was able to successfully find a suitable donor for transplantation and underwent the procedure at a tertiary center in the region. This transplantation was successful, and he was closely followed afterwards by both hematology teams. 

Sadly, a few months later, the patient developed a viral illness and deteriorated quickly and went into cardiac arrest at home. Many rounds of CPR were administered in the ambulance and at our hospital; however, the patient could not be resuscitated. No singular cause was identified for the sudden deterioration and the patient passed away in hospital. 

## Discussion

BPDCN is a rare and aggressive hematological cancer, with only 1000 cases reported in Europe and the US combined [9]. It was only recognized as a distinct category by the WHO in 2016 [10]. Cutaneous involvement is often the only initial sign of this malignancy, with up to 85% of people presenting with it [11], with a male to female predominance of about three to one [12]. The initial skin nodule is usually followed by further lesions or a quick deterioration of physical symptoms. This malignancy is rarely diagnosed clinically, especially in a presentation similar to our patient. In the absence of other features typical of malignancy, skin nodules associated with BPDCN may be attributed to other benign autoimmune causes, such as cutaneous lupus erythematosus [13] or systemic vasculitis [14], which also present with erythematous skin lesions, albeit more diffuse and less nodular than this malignancy. It is misdiagnosed as other more common malignancies, with one case report identifying a patient misdiagnosed and treated for follicular cell lymphoma, even with a biopsy conducted before diagnosis [15]. 

The cutaneous lesions associated with this malignancy closely resemble leukemia cutis, a skin manifestation of acute myeloid leukemia (AML). Leukemia cutis also tends to present on the face, trunk and extremities [16] as seen with this patient; however, immunohistochemical analysis was able to rule out AML and other leukemias associated with the development of leukemia cutis. An Epstein-Barr Virus fluorescence in situ hybridization (EBV-FISH) test was also performed during the initial workup for this malignancy, as the EBV is associated with various hematological malignancies, especially lymphomas (notably T-cell lymphomas, diffuse large B-cell lymphomas, and Burkitt's lymphoma) [17], which also present with cutaneous tumors in the form of plaques and patches. 

People are usually diagnosed based on an immunohistochemical analysis on a skin biopsy, with cell expression of CD123, CD4 and CD56 being diagnostic for this malignancy [3]. It is also associated with significant chromosomal alterations with hypodiploid or aberrant karyotypes seen frequently. However, these changes are not specific to the disease [18]. 

There is some contention over treatment for BPDCN, and both AML- and acute lymphoid leukemia (ALL)-type treatments are offered. Patients who have received ALL-type therapies have shown better outcomes, as reported by a large, multi-center analysis conducted by Taylor et al. [19]. This multicenter study, the only one of its kind for BPDCN, also highlights some characteristics associated with poorer outcomes. Older age of disease onset, particularly >60 years and abnormal karyotype were associated with the worst prognosis [19]. 

This study also highlights TdT as an important marker of disease prognosis, with TdT negativity associated with poorer outcomes [19]. Currently, there is little research on the role of tumor markers as prognostic factors for this malignancy, and large, multi-center analyses are needed to come to a definitive conclusion. However, the rarity of the disease and poor prognosis may make this challenging. 

While this malignancy’s response to cytotoxic agents is initially promising, it shows quick drug resistance and relapse [4], adding to its challenging nature. Delayed presentation, as with our patient, can further complicate management. Any lesion, whether surveyed in primary or secondary care, which persists despite initial treatment, or aggressively proliferates should raise immediate suspicion of a malignancy and should be biopsied immediately and any abnormal pathology urgently communicated with hematologists in secondary care. This case, where the patient presented without a previous malignancy or classical features pointing to one, highlights the importance of dermapathology in diagnosis. 

Despite the disease's difficult clinical management, recent advancements in targeted therapies may offer better outcomes for BPDCN in the future with one IL-3 recombinant fusion protein therapy targeting CD123 overexpression [1]. Tagraxofusp, which is a CD 123-directed cytotoxin has shown efficacy in reducing disease burden initially and after relapse [1,3]. This drug is currently not licensed for use in the United Kingdom and could not be used for our patient's management. Studies assessing its efficacy are still underway, however, it may significantly improve outcomes and potentially be used as an alternative to chemotherapy in the future. 

Relapse is common with BPDCN, with a median overall survival (OS) of up to 30 months without allogenic BMT (allo-BMT) [20]. This increases to about a 74-82% survival rate at three years with allo-BMT [20], however long-term prognosis is still poor. Allo-BMT is associated with its own complications, and not many people can qualify for it, as older people have other co-morbidities that may be a deterrent to the procedure. 

Even with an allogenic transplant, relapse is not uncommon, with about 30% of patients relapsing within the first year of transplant with typically bone marrow or CNS involvement [20]. Resistance to previously well-tolerated chemotherapy is a common feature of this malignancy [20]. Relapse is treated with additional cycles of intensive chemotherapy and after achieving partial remission, another allo-BMT may be offered with variable reported outcomes. 

## Conclusions

BPDCN is a hematological malignancy defined by abnormal precursor cell proliferation. It mainly affects males and older adults and has an aggressive clinical course. It is a challenging and rare diagnosis to make clinically; however, a quick skin biopsy of initial skin lesions and immunohistochemistry are crucial for timely diagnosis and management. 

This case underscores the importance of a multidisciplinary approach involving dermatologists, pathologists, and hematologists to optimize diagnosis and manage treatment. Continued research into BPDCN’s pathogenesis and therapeutic options is essential to improve patient outcomes for this rare and challenging malignancy. 
